# Comparison of the incidence, clinical features and outcomes of invasive candidiasis in children and neonates

**DOI:** 10.1186/s12879-018-3100-2

**Published:** 2018-04-24

**Authors:** Jen-Fu Hsu, Mei-Yin Lai, Chiang-Wen Lee, Shih-Ming Chu, I-Hsyuan Wu, Hsuan-Rong Huang, I-Ta Lee, Ming-Chou Chiang, Ren-Huei Fu, Ming-Horng Tsai

**Affiliations:** 1 Division of Pediatric Neonatology, Department of Pediatrics, Chang Gung Memorial Hospital, Taoyuan, Taiwan; 2 Division of Neonatology and Pediatric Hematology/Oncology, Department of Pediatrics, Chang Gung Memorial Hospital, No.707, Gongye Rd., Sansheng, Mailiao Township, Yunlin, Taiwan, Republic of China; 3 College of Medicine, Chang Gung University, Taoyuan, Taiwan; 4 Department of Nursing, Division of Basic Medical Sciences and Research Center for Industry of Human Ecology, Chang Gung University of Science and Technology, Chiayi, Taiwan; 5 Department of Medical Research, Taichung Veterans General Hospital, Taichung, Taiwan

**Keywords:** Bloodstream infection, Invasive candidiasis, Candidemia, Antifungal susceptibility, Mortality

## Abstract

**Background:**

Invasive candidiasis differs greatly between children and neonates. We aimed to investigate the different therapeutic approaches and their effects on treatment outcomes of these two groups.

**Methods:**

Episodes of neonatal invasive candidiasis were compared with non-neonatal pediatric episodes during a 12-year cohort study. Clinical isolates were documented by matrix-assisted laser desorption/ionization-time of flight mass spectrometry and DNA sequencing, and antifungal susceptibility testing was performed.

**Results:**

A total of 342 episodes of invasive candidiasis (113 neonatal and 229 non-neonatal pediatric episodes) in 281 pediatric patients (96 neonates and 185 children) were identified. *Candida albicans* was the most common pathogen causing invasive candidiasis in neonates and children (47.8% vs. 44.1%). The antifungal susceptibility profiles were not significantly different between neonates and children. More neonates received amphotericin B as therapy, whereas more children received fluconazole or caspofungin. Compared with children, neonates had a significantly longer duration of fungemia, higher rates of septic shock (34.5% vs. 21.8%; *P* = 0.013), sepsis-attributable mortality (28.3% vs. 17.5%; *P* = 0.024) and in-hospital mortality (42.7% vs. 25.4%; *P* = 0.004) than children. Independent risk factors for treatment failure of invasive candidiasis were septic shock (odds ration [OR] 16.01; 95% confidence interval [CI] 7.64–33.56; *P* <  0.001), delayed removal of intravenous catheter (OR 6.78; 95% CI 2.80–17.41; *P* <  0.001), renal failure (OR 5.38; 95% CI 1.99–14.57; *P* = 0.001), and breakthrough invasive candidiasis (OR 2.99; 95% CI 1.04–8.67; *P* = 0.043).

**Conclusions:**

Neonatal invasive candidiasis has worse outcomes than non-neonatal pediatric candidiasis. Neonatologists and pediatricians must consider age-specific differences when developing treatment and prevention guidelines, or when interpreting studies of other age groups.

## Background

*Candida* species are the fourth most common cause of nosocomial infection and are the leading cause of invasive fungal infection among hospitalized patients [1, 2]. Invasive candidiasis deserves greater attention because it is associated with a high mortality rate, especially in severely ill patients [3–5]. Recent population-based surveillance studies have shown an increased incidence of invasive candidiasis in intensive care units (ICUs) during the past decade [6, 7]. An increase of susceptible hosts who receive intensive care or immunosuppressive therapies and the widespread use of broad-spectrum antibiotics may account for the increase of invasive *Candida* infections [8–10]. Furthermore, uses of antifungal drugs such as azoles for prophylaxis and echinocandins for treatment are reported to be associated with a continuous shift from *C. albicans* to various non-*albicans Candida* species [11, 12].

The microbiological and clinical characteristics of invasive *Candida* infections vary widely among different geographic areas, patient characteristics and ages, and institutions [13, 14]. Although some studies concluded that the mortality of candidemia was higher in adults than in children [15], a recent study found a poor prognosis among infants (<1 year of age) and elderly patients (>60 years) [16]. Furthermore, we recently documented fungemia as an independent risk factor for treatment failure in the neonatal ICU (NICU) [17]. The reported incidence of candidemia in pediatric patients generally ranges between 0.21 and 10.5 cases per 1000 admissions [15, 18–20]; however, patients in the NICU, pediatric ICU (PICU), and pediatric wards were not studied as separate and distinct groups [15, 21]. In order to clarify and assess unique characteristics of invasive candidiasis in neonates and children, we compared the epidemiology and clinical features of *Candida* spp. identified by matrix-assisated laser desorption/ionization time-of-flight mass spectrometry (MALDI-TOF) causing invasive candidiasis in these two populations.

## Methods

We included all hospitalized patients in the Department of Pediatrics, Chang Gung Memorial Hospital (CGMH) from January 2004 through December 2015, for whom ≥1 blood culture and/or sterile site cultures were positive for Candida spp. and who had symptoms, signs, or laboratory findings consistent with fungal infection. We retrospectively reviewed electronic medical records for demographic, clinical and laboratory data for the onset of invasive candidiasis (defined as the day of blood or sterile site collection for culture), and we reviewed risk factors within the preceding 30 days, major comorbidities, complications of invasive candidiasis, treatments and outcomes. The study was approved by the Institutional Review Board and Human Research Ethics Committee of CGMH, and a waiver of informed consent for anonymous data collection was also approved.

Isolation and identification of all *Candida* spp. isolates in blood and sterile site cultures were performed using a standard API 32C AUX yeast identification kit (bioMérieux SA, Marcy l’Étoile, France) and chromogenic culture media (CHROMagar; Becton Dickinson and Company, USA). Since December 2013, we have used MALDI-TOF (Bruker Biotype, software version 3.0, Ewing, NJ, USA) and large-subunit (18S) ribosomal RNA gene D1/D2 domain sequencing to re-confirm all these species. Antifungal susceptibility was tested using the Clinical and Laboratory Standards Institute broth microdilution reference method [22]. For uncommon *Candida* spp., clinical breakpoints are undefined; therefore, isolates that showed minimum inhibitory concentrations (MICs) higher than the epidemiologic cutoff value were considered potentially resistant [23]. We excluded unidentified *Candida* species and selected only the first isolate recovered from the blood or sterile sites if a patient had several cultures that were positive for the same *Candida* spp.

### Definitions

Invasive candidiasis included candidemia and deep-seated candidiasis, which were defined as the recovery of a *Candida* species from blood or a sterile site, respectively [24, 25]. An episode of candidemia was considered to be catheter-related only if the catheter tip culture was positive for the same *Candida* spp. [26]. Episodes were considered to be separate if they occurred ≥1 month apart. Breakthrough invasive fungal disease was defined as candidemia or positive *Candida* spp. isolated from a sterile site in a patient who had undergone therapy or prophylaxis with any systemic antifungal drug for ≥3 consecutive days before the index blood culture [8, 27]. Invasive candidiasis-attributable mortality was defined when the patient died within 7 days after onset of invasive candidiasis or in the presence of persistent clinical sepsis or persistent candidemia, or if the patient died of candidemia associated complications [27, 28]. Combined with the antifungal susceptibility results, treatment failure was defined as an infection that led to attributable mortality or cases of persistent candidemia ≥7 days after initiation of effective antifungal therapy. Patient responses to antifungal therapy following invasive candidiasis were defined according to the consensus criteria of the Mycoses Study Group and the European Organization for Research and Treatment of Cancer [29].

### Statistical analysis

Clinical data were analyzed using SPSS version 18.0 (SPSS Inc., Chicago, IL, USA). Variables associated with invasive candidiasis in the NICU were compared with non-neonatal episodes. Univariate analyses were performed using Student’s *t*-test or non-parametric tests as appropriate (for continuous variables) or the chi-square or Fisher’s exact tests (for categorical variables). All tests were 2-tailed, and a *P* value of < 0.05 was considered significant. We performed multiple logistic regression analyses to identify clinical risk factors that were associated with treatment failure of invasive candidiasis. All risk factors that were significant at 0.10 in the univariate analysis were included in the corresponding multivariate analysis.

## Results

### Incidence and demographic data

Among a total of 20,545 neonatal admissions and 153,372 pediatric non-neonatal admissions (of which 14,018 were PICU admissions), there were a total of 342 invasive candidiasis episodes in 281 children that occurred during the study period; of these, neonatal episodes accounted for 113 episodes (33.0%, in 96 neonates). The incidence rates of invasive candidiasis in neonates (NICU) and non-neonatal pediatric patients were 26.9 episodes per 100,000 inpatient days and 32.6 episodes per 100,000 inpatient days, respectively. However, the incidence rate of invasive candidiasis in the PICU was 147.2 episodes per 100,000 inpatients days, which was significantly higher than the rates in the NICU and in the general pediatric ward (both *P* <  0.001). Overall, the annual incidence rates of invasive candidiasis did not change significantly throughout the study period and varied between 27.3 and 36.7 episodes per 100,000 inpatients days (data not shown).

**Fig. 1:**
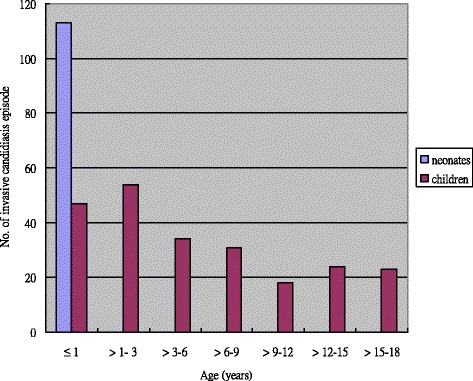
The age distribution of the occurrence of invasive candidiasis in children

Invasive candidiasis occurred in 152 boys (54.1% of all patients) and 129 girls. The mean age for non-neonatal pediatric patients was 6.2 ± 5.7 years (range, 3 months to 18 years). Overall, 214 (62.6%) episodes of invasive candidiasis occurred in children ≤3 years old (Fig. 1). Most of the invasive candidiasis episodes were primary bloodstream infections (228 episodes, 66.7%), followed by catheter-related bloodstream infections (69 episodes, 20.2%), and intra-abdominal infections (31 episodes, 9.1%). The sites of isolation and *Candida* species distributions were not significantly different between the neonatal and non-neonatal groups (Table 1), except that only two episodes in the NICU were caused by *C. tropicalis*. *C. albicans* was the most common *Candida* species that caused invasive candidiasis in children (45.3%, 155 episodes), followed by *C. parapsilosis* (27.8%, 95 episodes), *C. tropicalis* (6.4%, 22 episodes) and *C. glabrata* (6.1%, 21 episodes). Polyfungal isolates (i.e., two different *Candida* species yielded on cultures of blood samples that were obtained simultaneously) were recovered from three episodes, and the majority of ascites cultures (in 23 episodes, 74.2%) were polymicrobial isolates that also yielded gram-positive cocci, or aerobic and anaerobic gram-negative bacilli.

**Table 1:** Incidence and *Candida* spp. causing invasive candidiasis over a 12-year period in children

	Neonatal episodes (NICU)	Non-neonatal episodes	
		PICU	General wards
Total episodes	113 (33.0)	124 (36.2)	105 (30.7)
Incidence rate			
per 100,000 inpatient days	26.9	147.2	16.7
per 10,000 admissions	55.0	88.5	7.5
Pathogens			
*Candida albicans*	54 (47.8)	61 (49.2)	40 (38.1)
*Candida parapsilosis*	32 (28.3)	29 (23.4)	34 (32.4)
*Candida tropicalis*	2 (1.8)	13 (10.5)	7 (6.7)
*Candida glabrata*	10 (8.8)	7 (5.6)	4 (3.8)
Others	15 (13.3)	14 (11.3)	20 (19.0)
Sites of isolations			
Blood	103	118	97
Central venous catheter^a^	22	29	18
Abdomen	9	12	6
Urine^b^	4	7	2
Cerebrospinal fluid	2	2	1
Lung^c^	0	4	0

*NICU* neonatal intensive care unit, *PICU* pediatric intensive care unit

^a^Including Port-A catheter, Hickman catheter, and percutaneous inserted central venous catheter

^b^Suprapubic puncture for collection of urine in the NICU, and catheterization for collection of specimen in non-neonatal children

^c^Cultures from pleural fluid

### Risk factors

The majority of patients had multiple underlying illnesses and other risk factors that have been associated with invasive candidiasis (Table 2). The majority of neonatal invasive candidiasis cases occurred in very low birth weight infants (birth weight < 1500 g) (98 episodes, 86.7%), and the mean (SD) gestational age in this group was 27.8 ± 3.9 weeks. The most common predisposing factors were use of central intravenous catheter (CVC) (94.2%), use of broad-spectrum antibiotics (91.8%), stay in an ICU (69.3%), receipt of parenteral nutrition (64.6%), and underlying neurological sequelae (36.0%). For 282 episodes (82.4%), ≥ 4 risk factors and/or underlying illness were identified. However, the underlying illness and major predisposing factors were significantly different between neonatal invasive candidiasis and non-neonatal pediatric episodes. While neonates with invasive candidiasis were significantly more likely to have chronic lung disease and receive total parenteral nutrition, non-neonatal pediatric candidemia were more likely to occur in children with underlying neurological sequelae, cancer treated immunosuppressive agents, neutropenia and artificial devices other than CVC.

**Table 2:** Demographic and clinical characteristics of 342 episodes of invasive candidiasis in neonatal versus non-neonatal pediatric children

Characteristic	Neonatal episodes (total *n* = 113)	Non-neonatal pediatric episodes (total *n* = 229)	*P* value
Patient age, median (IQR)	27.0 (19.0–56.0) days	3.8 (1.2–10.6) years	–
Sex, male subjects/female subjects	68 (60.2)/45 (39.8)	110 (48.0)/119 (52.0)	0.039
Gestational age (weeks), median (IQR)	27.0 (25.0–29.0)	–	–
Underlying conditions^a^			
Congenital or genetic anomalies	9 (8.0)	25 (10.9)	0.447
Neurological sequelae	22 (19.5)	101 (44.1)	< 0.001
Cardiovascular disease	9 (8.0)	22 (9.6)	0.693
Chronic lung disease and/or pulmonary hypertension	60 (53.1)	33 (14.4)	< 0.001
Gastrointestinal sequelae	26 (23.0)	69 (30.1)	0.119
Renal sufficiency with/without dialysis	8 (7.1)	31 (13.5)	0.103
Hematological/Oncology cancer	0 (0)	48 (21.0)	< 0.001
Immunodeficiency	1 (0.9)	6 (2.6)	0.344
Autoimmune disease	0 (0)	8 (3.5)	0.056
Hepatic failure or cholestasis	4 (3.5)	9 (3.9)	1.000
Others^b^	1 (0.9)	1 (0.4)	–
Days of hospitalization before onset of invasive candidiasis, median (IQR)	26.0 (17.0–55.0)	29.5 (13.0–49.0)	0.818
Sequences of episodes			0.371
First episode	96 (85.0)	185 (80.8)	
Recurrent episode	17 (15.0)	44 (19.2)	
Associated risk factors^b^			
Receipt of systemic antibiotics^c^	106 (93.8)	208 (90.8)	0.407
Prior bacteremia^c^	43 (38.1)	126 (55.0)	0.004
Prior azoles exposure^c^	10 (8.8)	21 (9.2)	1.000
Presence of central venous catheter	108 (95.6)	214 (93.4)	0.625
Stay in an intensive care unit	113 (100)	124 (54.1)	< 0.001
Receipt of parenteral nutrition	105 (92.9)	116 (50.7)	< 0.001
Receipt of immunosuppressive drugs	3 (2.7)	65 (28.4)	< 0.001
Presence of artificial device other than central venous catheter	34 (30.1)	133 (58.1)	< 0.001
Prior surgery^c^	31 (27.4)	79 (34.5)	0.219
Neutropenia^d^	12 (10.6)	70 (30.6)	< 0.001

All data were expressed as number (percentage %), unless indicated otherwise; *IQR* interquartile range

^a^Indicated the presence of underlying condition or risk factor at onset of invasive candidiasis, and most episodes occurred in patients with > 1 underlying condition or risk factor

^b^One neonatal episode occurred in a patient with epidermolysis bullosa, and one non-neonatal pediatric episode occurred in a patient with diabetes mellitus

^c^Within 1 month prior onset of invasive candidiasis

^d^Absolute neutrophil count ≤500 cells/μL

### Clinical presentations

No patient developed endophthalmitis, endocarditis, or osteomyelitis that was clinically evident, but five had CNS infection and four had an obstructing renal fungus ball during the follow-up period. In addition, 31 had intraabdominal abscesses or peritonitis and four patients had positive *Candida* isolates from pleural fluids. 36.8% of invasive candidiasis presented with severe sepsis, and 26.0% had septic shock at the onset of sepsis. After effective antifungal treatment, 17.8% had progressive and deteriorated candidiasis, and 14 (4.1%) had disseminated candidiasis. Neonates with invasive candidiasis had significantly higher severity of illness than children based on the surrogate marker of severe sepsis and septic shock (Table 3).

**Table 3:** Clinical features, treatment and outcomes of invasive candidiasis in neonatal episodes versus non-neonatal pediatric episodes

	Neonatal episodes (total *n* = 113)	Non-neonatal pediatric episodes (total *n* = 229)	*P* value
Clinical features			
Severe sepsis	55 (48.7)	71 (31.0)	0.002
Septic shock	39 (34.5)	50 (21.8)	0.013
Progressive and deteriorated candidiasis^a^	27 (23.9)	34 (14.8)	0.050
Disseminated candidiasis^b^	5 (4.4)	9 (3.9)	0.828
Breakthrough invasive candidiasis	10 (8.8)	31 (13.5)	0.288
Duration of candidemia and/or persistent invasive fungal infection			
Days, median (interquartile range)	3.0 (1.0–6.0)	1.0 (1.0–5.0)	0.033
≤2 days	48 (42.5)	131 (57.2)	
3–7 days	46 (40.7)	59 (25.8)	
≥8 days	19 (16.8)	39 (17.0)	
Antifungal regimens for treatment			< 0.001
Fluconazole/Voriconazole	34 (30.1)	97 (42.4)	
Amphotericin B	50 (44.2)	47 (20.5)	
Echinocandin	20 (17.7)	76 (33.2)	
Combination antifungal treatment	6 (5.3)	2 (0.9)	
None	3 (2.7)	7 (3.1)	
Antifungal treatment within 24 h	36 (31.8)	105 (45.9)	0.014
Duration between onset of invasive candidiasis and initiation of antifungal agents, mean ± SD (days)	2.1 ± 1.3	1.7 ± 1.4	0.009
Total treatment duration (days), mean (range)	17.5 (2.0–46.0)	18.9 (1.0–68.0)	0.113
Removal of central venous catheter within 3 days of onset	34/108 (31.5)	73/214 (34.1)	0.622
Treatment outcomes			
Responsiveness after initiation of antifungal treatment^c^			0.157
Within 72 h	39 (34.5)	96 (41.9)	
4–7 days	17 (15.0)	47 (20.5)	
More than 7 days	21 (18.6)	41 (17.9)	
Treatment failure	36 (31.0)	45 (19.7)	0.015
Modification of antifungal treatment	44 (38.9)	107 (46.7)	0.203
Invasive candidiasis attributable mortality	32 (28.3)	40 (17.5)	0.024
In-hospital all-cause mortality	41/96 (42.7)	47/185 (25.4)	0.004

All data were expressed as number (percentage %), unless indicated otherwise

^a^Defined as candidemia episodes with more disseminated candidiasis and/or progressive multi-organ failure even after effective antifungal agents

^b^Indicated positive *Candida* isolates recovered from more than two sterile sites, in addition to primary bloodstream infection

^c^Responsiveness was defined according to the consensus criteria of the Mycoses Study Group and European Organization for Research and Treatment of Cancer [29]

More than half of the episodes (180 episodes, 52.6%) were characterized by fungemia or persistent invasive candidiasis of > 1 day’s duration, whereas 165 (48.2%) and 78 (22.8%) were characterized by fungemia or persistent invasive candidiasis of ≥3 days and ≥ 7 days, respectively. The mean duration of fungemia was 4.4 days (± 6.2 days). The longest duration of fungemia was 34 days, which occurred in a patient who had end-stage renal disease and who received long term hemodialysis.

### Treatment and outcomes

Of the 342 episodes, 332 (97.1%) were treated with an antifungal agent, and there were 41 episodes of breakthrough invasive candidiasis. Ten episodes (2.9%) were not treated because of the patient’s death before or at the time of the diagnosis was established. Antifungal therapy was initiated after a mean of 1.81 days (range, 0–6) following the acquisition of the first diagnostic blood and/or sterile site culture and was significantly later in neonates than in children (2.1 ± 1.3 vs. 1.7 ± 1.4 days, *P* = 0.009). The mean duration of all antifungal therapy per episode was 18.5 days (range, 1–68). Of those 332 episodes for which an antifungal agent was used, 151 episodes (45.5%) had modification of the antifungal regimens during the treatment course, mainly due to the patient’s poor response to initial antifungal therapy (101 episodes, 66.9%), suspicious antifungal resistance after confirmation of *Candida* spp.(36 episodes, 23.8%), or no reason was documented (14 episodes, 9.3%). Among the antifungal regimens for treatment, fluconazole was the most commonly prescribed initially (62.3%), followed by amphotericin B (24.7%) and caspofungin (4.5%). However, the final treatment regimens were fluconazole/Voriconazole (39.5%), amphotericin B (29.2%) and echinocandin (28.9%), with significant differences between neonates and children (Table 3).

**Fig. 2:**
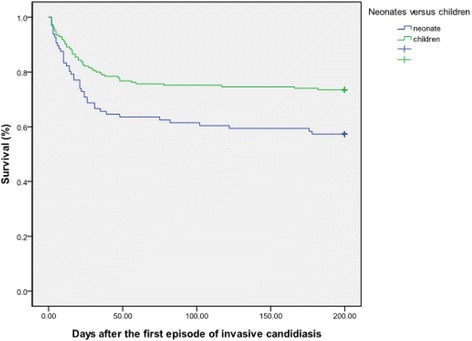
Survival following the first episode of invasive candidiasis in neonates when compared with children by the Kaplan-Meier method (log-rank test = 0.005)

Catheter removal was done within 3 days after illness onset in only one-third of patients with invasive candidiasis (107 episodes, 32.2%), and in 22 episodes, the candidemia resolved only after removal of the intravenous catheter. Neonates with invasive candidiasis had a longer period of fungemia than children, and a higher rate of treatment failure was also noted (31.0% vs. 19.7%, *P* = 0.015) (Table 3). Invasive candidiasis in neonates was associated with a significantly higher rate of sepsis-attributable mortality than that in children (28.3% vs. 17.5%, *P* = 0.024). After invasive candidiasis, neonates had a higher rate of in-hospital mortality than children (42.7% vs. 25.4%, *P* = 0.004, and *P* = 0.005 by log rank test [Fig. 2]).

### Susceptibility studies

In vitro susceptibility to various commonly prescribed antifungal agents in our hospital was determined for 295 isolates (Table 4). The rate of fluconazole-R or S-DD *Candida* was 14.6% (43 of 295 isolates) overall. The antifungal susceptibility profiles of *Candida* spp. in neonates were not significantly different between those in non-neonatal pediatric episodes. No trend toward higher minimum inhibitory concentrations was noted when earlier isolates (i.e., isolates recovered during 2004–2009) were compared with those obtained later (i.e., those recovered during 2010–2015).

**Table 4:** In vitro susceptibility to various antifungal agents of selected Candida species causing invasive candidiasis in neonatal versus non-neonatal pediatric patients

	All *Candida* (total *n* = 295)			*C. albicans*		*C. parapsilosis*		Other *Candida* spp.	
	All episodes	Neonates	Children	Neonates	Children	Neonates	Children	Neonates	Children
Fluconazole									
Susceptible	251 (85.1)	87 (83.7)	164 (85.9)	47 (97.9)	73 (94.8)	30 (100)	59 (100)	10 (38.5)	32 (58.2)
S-DD or R	44 (17.5)	17 (16.3)	27 (14.1)	1 (2.1)	4 (5.2)	0 (0)	0 (0)	16 (61.5)	23 (41.8)
Voriconazole									
Susceptible	262 (88.8)	97 (93.3)	165 (86.4)	47 (97.9)	73 (94.8)	30 (100)	59 (100)	20 (76.9)	33 (60)
S-DD or R	33 (11.2)	7 (6.7)	26 (13.6)	1 (2.1)	4 (5.2)	0 (0)	0 (0)	6 (23.1)	22 (40)
Amphotericin B									
Susceptible	292 (99.0)	104 (100)	188 (64.6)	48 (100)	77 (100)	30 (100)	59 (100)	26 (100)	52 (94.5)
S-DD or R	3 (1.0)	0 (0)	3 (1.6)	0 (0)	0 (0)	0 (0)	0 (0)	0 (0)	3 (5.5)
Micafungin									
Susceptible	291 (98.6)	102 (98.1)	189 (99.0)	48 (100)	76 (98.7)	28 (93.3)	58 (98.3)	26 (100)	55 (100)
S-DD or R	4 (1.4)	2 (1.9)	2 (1.0)	0 (0)	1 (1.3)	2 (6.7)	1 (1.7)	0 (0)	0 (0)
Caspofungin									
Susceptible	293 (99.3)	104 (100)	189 (99.0)	48 (100)	77 (100)	30 (100)	59 (100)	26 (100)	53 (96.4)
S-DD or R	2 (0.7)	0 (0)	2 (1.0)	0 (0)	0 (0)	0 (0)	0 (0)	0 (0)	2 (3.6)

All data were expressed as number (percentage %), unless indicated otherwise

*S-DD* susceptible-dose dependent, *R* resistant

### Independent risk factors for treatment failure

Except for underlying renal failure that required hemodialysis, none of underlying chronic comorbidities were associated with treatment failure. Treatment failure was not associated with any specific *Candida* species that caused invasive candidiasis. After multivariate logistic regression analysis (Table 5), the independent risk factors for treatment failure of invasive candidiasis included septic shock (odds ratio [OR]: 16.01; 95% confidence interval [CI]: 7.64–33.56; *P* <  0.001), delayed removal of intravenous catheter (after 3 days of disease onset) (OR: 6.78; 95% CI: 2.48–18.52; *P* <  0.001), underlying renal failure with/without hemodialysis (OR: 5.38; 95% CI: 1.99–14.57; *P* = 0.001), and breakthrough invasive candidiasis (OR: 2.99; 95% CI: 1.04–8.67; *P* = 0.043).

**Table 5:** Risk factors for treatment failure in pediatric invasive candidiasis by univariate and multivariate analysis

Risk factors	Univariate analysis			Multivariate analysis	
	Treatment success (total *n* = 261)	Treatment failure (total *n* = 81)	*P* value	Adjusted OR (95% CI)	*P* value
Neonates vs. children					
Neonates	77 (29.5)	36 (44.4)	0.015	1.96 (0.91–4.23)	0.087
Children	184 (70.5)	45 (55.6)		1 (reference)	
Initiation of antifungal agents within 24 h	100 (38.3)	41 (50.6)	0.054	1.58 (0.79–3.16)	0.540
Breakthrough invasive candidiasis	24 (9.2)	17 (21.0)	0.010	2.99 (1.04–8.67)	0.043
Septic shock at onset	34 (13.0)	55 (67.9)	< 0.001	16.01 (7.64–33.56)	< 0.001
Underlying renal failure with/without hemodialysis	20 (7.7)	19 (23.5)	< 0.001	5.38 (1.99–14.57)	0.001
Delayed catheter removal > 3 days after illness onset	154 (59.0)	71 (87.7)	< 0.001	6.78 (2.48–18.52)	< 0.001
Treatment regimens			0.001		
Fluconazole	109 (40.6)	22 (27.2)		1 (reference)	
Amphotericin B	73 (28.0)	24 (29.6)		1.53 (0.70–3.33)	0.289
Echinocandin	73 (28.0)	23 (28.4)		1.04 (0.47–2.31)	0.933
Combination therapy	6 (2.3)	2 (2.5)		1.76 (0.25–12.3)	0.570
No antifungal treatment	0 (0)	10 (12.3)		10.07 (1.6–64.7)	< 0.001
Pathogens			0.729		
*Candida albicans*	121 (46.4)	34 (42.0)			
*Candida parapsilosis*	71 (27.2)	24 (58.0)			
*Candida tropicalis*	15 (5.7)	7 (8.6)			
*Candida glabrata*	17 (6.5)	4 (4.9)			
Other *Candida* spp.	36 (13.8)	12 (14.8)			
Infectious source			0.358		
Primary bloodstream infection	169 (64.8)	59 (72.8)			
Catheter-related bloodstream infection	59 (22.6)	10 (12.3)			
Intra-abdominal	22 (8.4)	9 (11.1)			
Urological	4 (1.5)	1 (1.2)			
Lung	3 (1.1)	1 (1.2)			
Meningitis	4 (1.5)	1 (1.2)			

## Discussion

The epidemiology and choice of therapy for candidemia or invasive candidiasis are rapidly changing, and vary greatly in different settings, age groups, or geographic areas [15, 28, 30]. The crude mortality rates are generally lower in younger (pediatric) than older (adult) patients with candidemia regardless of the *Candida* species [15, 30]. A recent prospective multicenter surveillance study of candidemia has showed a higher 30-day mortality rate in neonates with candidemia than children (40% vs. 28%, *P* = 0.02) [31]. Our study further demonstrated that neonatal invasive candidiasis has worse responsiveness to antifungal therapy, more prolonged fungemia, more likely to have severe sepsis and septic shock, and higher rates of sepsis attributable mortality and in-hospital mortality.

Several factors can affect the treatment outcomes of candidemia, including underlying chronic comorbidities, microbiological factors, treatment policies and timely administration of antifungal agents, illness severity, and treatment with an infected catheter in situ or removal [3, 14, 32–35]. The underlying illness and predisposing factors for neonatal candidiasis and non-neonatal pediatric candidiasis are basically different. We found extreme prematurity and related comorbidities, including use of total parenteral nutrition and underlying chronic lung disease to be the major predisposing factors for neonatal candidemia [31]. In non-neonatal pediatric candidiasis, underlying chronic conditions, especially neurological sequelae, hemodialysis, hematological/cancer patients on immunosuppressive treatment and resulting neutropenia accounted for the majority of the susceptible hosts. Administration of antifungal agents is less frequently delayed in the non-neonatal pediatric setting because the underlying chronic comorbidities would remind clinicians of the possibility of opportunistic infections. Furthermore, we identified incidental cases of congenital candidemia without any of these underlying illnesses or risk factors, which has rarely been reported [36].

Because most of our *Candida* isolates were sensitive to all commonly prescribed antifungal agents, treatment failures most likely were caused by the failure of infectious sources control and underlying illness. Our results are similar to those of Grim et al. [37], who concluded that a high mortality rate among patients with candidemia (34% mortality within 30 days), with underlying cirrhosis and HIV infection, and increased illness severity were the independent risk factors, despite timely receipt of appropriate antifungal therapy. We documented that removal of an infected catheter was an important factor for successful treatment of invasive candidiasis [38, 39]. Although breakthrough candidemia was not associated with nonsusceptible fluconazole isolates [8, 40], it was independently associated with treatment failure and also more prolonged fungemia in our cohort.

This study included all episodes of invasive candidiasis in children [41]. Although some episodes were blood culture-negative intra-abdominal candidiasis and some were *Candida* spp. positive only in the pleural fluid, all cases of invasive candidiasis were evaluated by the infection specialist and were found to require treatment, unless mortality preceded the diagnosis of invasive fungal infection. Currently no clinical study has assessed the need to treat *Candida* peritonitis [42, 43], but we excluded cases of probable *Candida* colonization [44]. Furthermore, the high proportion of mixed-flora peritonitis is one limitation in this study because the pathogenic role of *Candida* in this polymicrobial form of infection is a matter of debate. However, many experts still consider positive *Candida* cultures from intraabdominal fluid in patients with peritonitis to be clinically significant even in the presence of concomitant bacterial growth [45]. Antifungal therapy is recommended by the European Society of Clinical Microbiology and Infectious Diseases and the Infectious Disease Society of America guidelines on management of complicated intra-abdominal abscess or peritonitis that is positive for *Candida* spp. [46, 47].

Recent studies described an overall mortality of 17.2–46.2% among children with invasive candidiasis [15, 19–21, 30, 48–50], and mortality attributable to candidemia has been reported to be 12–22% [15, 30, 48–50]. Some *Candida* species were associated with worse outcomes. For example, *C. tropicalis*, *C. krusei* and *C. glabrata* related candidemia are associated with higher mortality rates than *C. parapsilosis* related candidemia in adults [28, 48]. However, our cohort did not include enough patients to support any firm conclusions in the pediatric settings. We found the choice of antifungal therapy did not appear to have a significant impact on treatment result [51–53]. In our cohort, patients who received fluconazole alone had a more favorable outcome than did patients who received other regimens, reflecting a bias toward the administration of fluconazole therapy to patients who were less ill.

The strengths of our study include the systematic identification of specific species of *Candida* isolates, the systemic collection of clinical data, and the fact that this is the first report that focuses specifically on the difference between neonatal and non-neonatal pediatric settings. However, there were some limitations in this study. First, this study was a retrospective study and conducted in a single center. Therefore, extrapolation of the findings to other institutions must be done cautiously. Second, severity of illness was not documented because the scoring systems were different in the neonatal and non-neonatal period. Third, *Candida* surveillance cultures were not obtained, and there were no data regarding prior colonization [19]. We failed to document all the risk factors for invasive candidiasis in this study. Furthermore, the policies regarding antifungal therapy may be changed over such an extended study period in our non-interventional study, which should be considered as a description of clinical practice only.

## Conclusions

In conclusion, this study demonstrates some significant differences of invasive candidiasis between neonates and children. Neonatologists and pediatricians must consider age-specific differences when developing treatment and prevention guidelines, or when studies of other age groups are interpreted. Furthermore, because the clinical signs of invasive candidiasis are not specific and early microbiological documentation remains a major challenge, intensive research dedicated to the development of alternative tools for early diagnosis of invasive candidiasis is urgently warranted.

## Abbreviations

BPD: Bronchopulmonary dysplasia
BSI: Bloodstream infection
CDC: Centers for Disease Control and Prevention
CI: Confidence interval
CRP: C-reactive protein
CVC: Central venous catheter
ESBL: Extended-spectrum β-lactamase
GBS: Group B streptococcus
GPC: Gram-positive cocci
IQR: Interquartile range
MALDI-TOF: Matrix-assisted laser desorption ionization time-of-flight
NEC: Necrotizing enterocolitis
NICU: Neonatal intensive care unit
NTISS: Neonatal Therapeutic Intervention Scoring System
OR: Odds ratio
PVL: Periventricular leukomalacia
RDS: Respiratory distress syndrome
TPN: Total parenteral nutrition
VAP: Ventilator associated pneumonia
